# A damping circadian clock drives weak oscillations in metabolism and locomotor activity of aphids (*Acyrthosiphon pisum*)

**DOI:** 10.1038/s41598-017-15014-3

**Published:** 2017-11-02

**Authors:** Katharina Beer, Jens Joschinski, Alazne Arrazola Sastre, Jochen Krauss, Charlotte Helfrich-Förster

**Affiliations:** 1 Neurobiology and Genetics, Biocenter, University of Würzburg, Würzburg, Germany; 2 Animal Ecology and Tropical Biology, Biocenter, University of Würzburg, Würzburg, Germany

## Abstract

Timing seasonal events, like reproduction or diapause, is crucial for the survival of many species. Global change causes phenologies worldwide to shift, which requires a mechanistic explanation of seasonal time measurement. Day length (photoperiod) is a reliable indicator of winter arrival, but it remains unclear how exactly species measure day length. A reference for time of day could be provided by a circadian clock, by an hourglass clock, or, as some newer models suggest, by a damped circadian clock. However, damping of clock outputs has so far been rarely observed. To study putative clock outputs of *Acyrthosiphon pisum* aphids, we raised individual nymphs on coloured artificial diet, and measured rhythms in metabolic activity in light-dark illumination cycles of 16:08 hours (LD) and constant conditions (DD). In addition, we kept individuals in a novel monitoring setup and measured locomotor activity. We found that *A*. *pisum* is day-active in LD, potentially with a bimodal distribution. In constant darkness rhythmicity of locomotor behaviour persisted in some individuals, but patterns were mostly complex with several predominant periods. Metabolic activity, on the other hand, damped quickly. A damped circadian clock, potentially driven by multiple oscillator populations, is the most likely explanation of our results.

## Introduction

The environment cycles in a daily manner due to the earth’s rotation around its own axis. In order to cope with these rhythmic changes, organisms rely on endogenous circadian clocks. Clocks drive behavioural and physiological rhythms with periods of approximately 24 h, and thus align the organism with the environmental rhythm^1,2^. These rhythms persist even without environmental time cues (Zeitgebers) such as light or temperature^3^. Therefore, circadian clocks enable organisms not only to react to, but also to predict environmental oscillations, which may be adaptive^4^. Hence, circadian clocks play an important role in coping with daily environmental changes.

Circadian timekeeping might also be involved in the photoperiodic calendar and control of diapause induction, although this hypothesis is still under discussion^5,6^. Bünning proposed that the circadian clock forms the basis of photoperiodism^7^, and this idea was later formalized as external^8^ and internal^9^ coincidence models. In contrast, Lees did not observe any circadian pattern in the photoperiodic reaction of aphids and excluded therefore the involvement of a circadian oscillator. He proposed an hourglass mechanism instead, in which some biochemical product accumulates during night, so that only sufficiently long nights can trigger a photoperiodic response^10,11^. But some phenomena of long night experiments cannot be explained by an hour-glass mechanism, and a model with a damping circadian oscillator describes diapause induction better^12^. By rephrasing the hour-glass mechanism as a damping oscillator, the model unites these apparently contrary views^13^. However empirical data demonstrating the actual damping of circadian oscillations is largely lacking.

Aphids are central to the discussion about photoperiodic clock involvement. During summer the insect produces offspring via parthenogenesis, thereby ensuring quick population growth. In autumn sexual morphs are induced, which produce cold resisting eggs able to survive winter^14^. The switch in reproductive modes is induced by shortening day length^15,16^ and, to a lesser extent, drops in temperature. This extraordinary phenotypic plasticity allows aphids to cope with seasonal changes, and is probably one reason for their global distribution. Due to the rapid population growth in summer, various species are classified as crop pests^17^, and climate change with associated shifts in phenology (seasonal timing) will likely exacerbate the pest pressure of aphids^18^. Thus, aphids are well-suited models for circadian and seasonal time-keeping, not only due to their long research history, but also because the timing matters for applied pest management.

There is ongoing interest in the circadian clock of aphids. Several behavioural studies suggest the existence of a functional circadian clock in sexual as well as in parthenogenetic aphid forms of various aphid species^19,20^, although the experimental protocols did not allow to investigate damping of the clock. More recently, two studies identified putative clock genes and the location of clock gene expressing neurons in the pea aphid *Acyrthosiphon pisum* (*Harris*)^21,22^. Unfortunately the challenge to uncouple the aphid’s activity from the plant’s influence remained unattended in most studies. Recently we described the first daily rhythms of aphids completely independent of their host plant^23^, but have so far not monitored aphid rhythms in constant darkness for methodological reasons.

In the present study we investigate two different outputs of the circadian clock, namely locomotor activity and metabolic activity independently of the host plant. We show that the aphid clock drives weak, but stable, circadian output rhythms in locomotion, whereas oscillations in metabolic activity dampen quickly in constant conditions.

## Material and Methods

In order to investigate the circadian behaviour of aphids, we performed two experiments: First, we supplied a coloured diet and tested for rhythms in honeydew excretion. Secondly, we applied a novel method that allows constant locomotor activity monitoring of sap-sucking insects. All experiments were performed independently of the host plant on an artificial diet (based on Febvay, *et al*.^24^, 20% (w/v) sugar). The aphids were reared in a small containment and separated from the food source by a parafilm M membrane (BEMIS COMPANY INC., USA)^25^, so they could access the diet from below by piercing the parafilm. The artificial diet was sterile filtrated through a 0.45 µm Minisart® syringe filter (Sartorius, Germany), and all materials that came in contact with the diet were sterilized before use. For the metabolic activity experiments we reduced the diet by eight ingredients, reordered the ingredients list, and supplemented it with 1.25 mg/ml Brilliant blue FCF^26^.

For all experiments we used an asexual *Acyrthosiphon pisum* line (LL01), a green alfalfa biotype from the Lusignan area that was kindly provided by G. Febvay (INRA Lyon, France). Stock cultures were kept on *Pisum sativum* (L.) var. Fuego plants in climate chambers (Sanyo/Panasonic MLR-H series; 18 ± 0.5 °C, 80% ± 10% RH, LD 16:08). All statistical tests were performed in R version 3.1.1^27^.

### Metabolic activity

We measured metabolic activity simultaneously under LD 16:08 and constant darkness for eight days. We conducted the experiment in climate chambers (Sanyo MLR-352H) at 18 °C and 70% humidity under a 15000 lux fluorescent light source. We placed 100 adult aphids on freshly cut broad bean leaves. On the next day, we discarded the adults, placed 120 nymphs individually in petri dishes (∅35 mm) and fed each with 500 µl coloured artificial diet. 60 aphids were moved into DD after five hours (Zeitgeber time (ZT) 12 = 12 hours after lights on), whereas the other 60 nymphs remained in LD, but at a reduced light intensity (7200 lux). At ZT 21 (i.e., three hours before lights-on), we counted and marked all honeydew drops which have been produced so far. Thus, the aphids in DD were given 9 hours to locate the food source before the start of measurements. We then counted honeydew every 3 hours (8 measurements per day). In addition, we counted all exuviae, as moulting individuals are not expected to produce honeydew.

Four observers have been involved in taking measurements. The time schedule was allocated in a non-random order, so that each observer occupied a different time slot every day. We always measured the replicates in the same order and timeframe. Measurements in the light phase were conducted under room light while measurements in the dark phase were made under red LED light. The excreted blue honeydew drops were equally visible under both light conditions.

The first half of the replicates in the LD treatment was removed from the experiment, because they were accidentally taken into room-light during lights-out in the first night. Hence, sample sizes were 30 in LD and 60 in DD. Because we did not renew the diet during the experiment in order to reduce disturbance, petri dishes became contaminated during the experiment and were subsequently removed. Thus, sample sizes decreased over time, with 80% of the samples remaining for at least 5 days. In LD sample size decreased in total from 30 to 14, and in DD from 60 to 17.

Only 3.4% of the aphids produced more than one honeydew drop in the observed time interval of three hours, so we treated the response as binomial. To test for metabolic rhythmicity, we applied a generalized linear mixed-effects model with binomial error distribution^28^ with the fixed factors “time of day” and “day”, and with the random term (“time of day” | “ID”). For the DD treatment, we expected the effect to dampen over the course of the experiment, so we incorporated a change in effect size over time. Hence, we used the fixed factor “time of day”, interacting with the continuous variable “time since start”, and the random term (“time of day” | ID). We checked all models for overdispersion. Because p-values are not reliable for GLMMs^29^, we report only confidence intervals^30^ for all models.

Honeydew production was infrequently interrupted by moulting, usually three times per individual. Thus, the aphids were in the final larval stage at the end of the experiment. To test whether moulting individuals produce less honeydew, we pooled all time points and applied a chi-square test of goodness of fit (2 × 2 contingency table: drop production vs moulting). Because moulting individuals indeed produced less honeydew (see results), we repeated the analysis with a reduced dataset that included only non-moulting individuals (6% of all measurements removed). In addition, we tested whether moulting itself was rhythmic, using the same model as for honeydew production in LD.

### Locomotor activity

**Figure 1:**
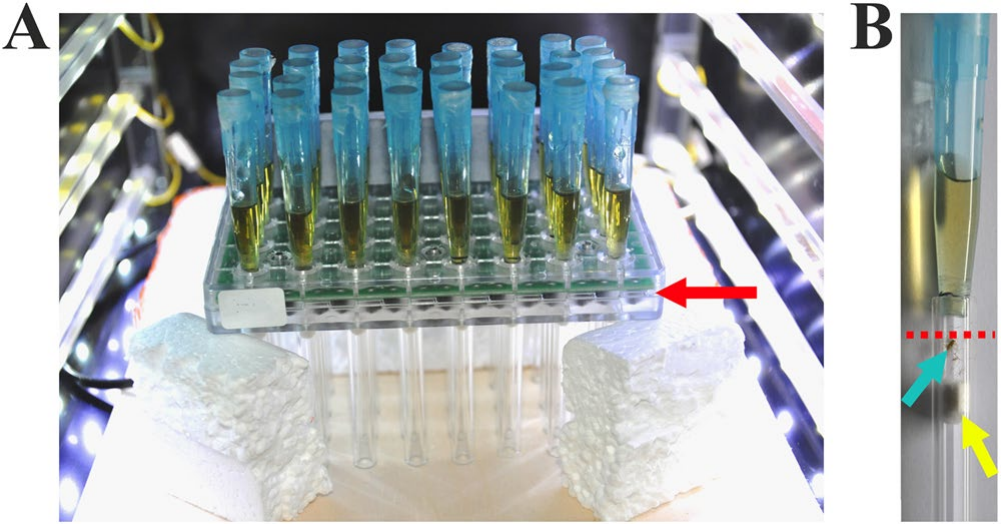
Locomotor activity monitoring set up. (**A**) Monitor (DAM2, Trikinetics) placed horizontally with illumination from the sides. 32 IR-beams on the monitor detect movements of individual aphids in glass tubes (**B**) with blue food tubes on top. Aphid (blue arrow) in a monitoring glass tube hanging from the food tube. The roaming space was limited by a cotton plug (yellow arrow). IR-beam (IR-beam level defined by the position of IR-beam emitter and sensor in the monitor is indicated by the red arrow and beam line crossing the monitoring tube is indicated by the dotted line) is positioned on the mid plane of the monitor and detects moving aphids.

For the locomotor activity experiments we used one day old offspring of aphids that were raised on plants and then kept on artificial diet for one day in order to control for age. We adapted the DAM2 (Drosophila Activity Monitor) (Trikinetics, USA), which monitors locomotor activity via an infrared-light barrier (IR-beam), to aphid specific requirements (Fig. 1). Aphids were provided with food ad libitum in a shortened micropipette tip (volume 1000 µl) on top of the monitor tube (∅7 mm) while the activity monitors were located horizontally. We positioned the IR-beam directly under the micropipette tip and limited the animals roaming space to 1 cm. The setup allowed monitoring 32 aphids simultaneously. All animals were entrained as nymph to an LD 16:08 regime (16 hours light and 8 hours darkness) for at least 12 days. A light intensity of 200–400 lux was produced by white light LEDs (depending on the position of the monitor tube) in the light phase and 0 lux in the dark phase while temperature and humidity were kept constant (18 ± 0.5 °C, 80% ± 10% RH) in the climate chamber (Percival INTELLUS, CLF Plant Climatics GmbH, 86637 Wertingen, Germany). On day 13 one treatment group (32 aphids) was released into constant DD conditions whereas another group of 32 aphids received LD conditions throughout their life.

Activity data was recorded in beam crosses per minute and evaluated with the ImageJ software plugin ActogramJ^31^ (Fiji ImageJ Version 1.49, © Wayne Rasband, National Institutes of Health, USA). We calculated the average day activity profile and mean activity in light and dark phase with the activity data of several consecutive days in Microsoft Excel (2013 Microsoft Office) and day activity was tested with Wilcoxon rank sum test. We excluded days 8–12, as earlier trials have shown that aphids undergo their last moulting in this time. It was impossible to appoint the aphids to either nymph or adult status reliably during this period without interrupting data acquisition.

We tested for rhythmicity in activity of individual adult aphids across at least five consecutive days in entraining (LD) and free-running (DD) conditions with Lomb-Scargle (LS) and chi-square periodogram (CS) analysis (period 1140–1740 minutes; smoothing factor 10 (only in chi-square method); p-level 0.05). In case of several narrow spikes that barely exceeded the significance level, we appointed the periodogram as false positive and the animals as arrhythmic (see definition in^32^). Differences in the groups “arrhythmic”, “rhythmic” (with simple circadian rhythms) and “complex” (with complex rhythms) between LD and DD conditions were tested with Fisher’s exact test. Individuals that did not undergo all four moultings were excluded from analysis.

### Data availability

All data generated or analysed during this study are included in this published article (and its Supplementary Information files). This includes R - scripts for analysis and figure reproduction of the metabolic activity experiment.

## Results

### Metabolic activity: Bimodal rhythmicity profile under LD and DD

We monitored metabolic activity of individual aphids over a period of eight days, by counting the number of exuviae and of visibly coloured honeydew drops every three hours. The aphids moulted three times during the eight days of measurement, on average after 24 (±3.25), 71 (±3.21) and 127 (±4.19) hours (days 1, 3 and 6). In total, we observed 252 of the expected 270 occurrences of moulting, because some aphids died during the course of the experiment. These occurrences of moulting suppressed honeydew excretion, because only 13.75% of the moulting individuals in LD produced honeydew drops, compared to 33.76% of the non-moulting individuals (χ² = 13.75, p < 0.001). In DD 18.13% of the moulting, and 29.41% of the non-moulting individuals excreted honeydew (χ² = 9.38, p < 0.01). We therefore excluded moulting individuals from the analysis of honeydew excretion. Moulting itself was not rhythmic, neither in LD nor in DD (not shown).

**Figure 2:**
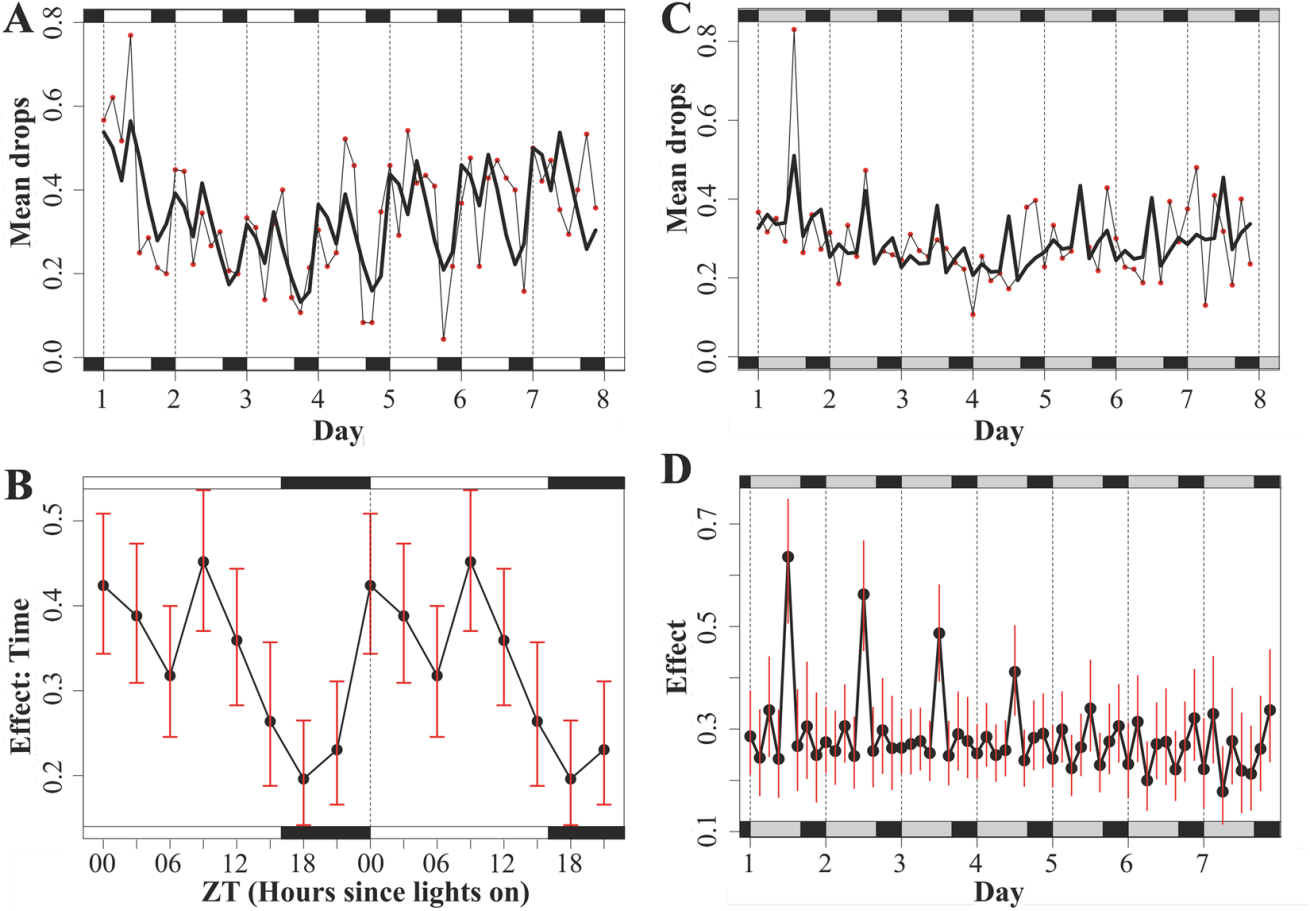
Rhythms in honeydew excretion. (**A**) Honeydew production of 30 aphid nymphs, kept under LD 16:08 for seven consecutive days. Red dots mark the mean number of produced honeydew drops during a time interval of three hours. The bold black line describes model fit to the data. (**B)** Double plotted model results for the “Time” factor (average day for an average individual) of metabolic activity (mean of 7 days in LD 16:08) with confidence intervals (red). (**C)** Red marked values are the mean number of honeydew drops produced during seven consecutive days in DD. Under these conditions a model that includes damping (“time of day” and “day” interaction) yielded the best fit to the data (bold black line). (**D)** Effect plot of metabolic rhythm during seven days in DD with confidence intervals (red). Lighting conditions during the experiment are indicated above and below in the environmental bars. White: light, black: darkness, grey: the light phase that was present before switching to DD.

The aphids which did not moult produced on average 2.7 drops per day in LD and 2.4 drops per day in DD (1230 drops in total). The data for LD conditions was best described with a model that only incorporates ‘time of day’ and ‘day’ (Fig. 2A; AIC = 1736), because the inclusion of a damping component (‘time of day’ * ‘time’ interaction) estimated more parameters but did not significantly improve the model fit (AIC = 1752). Using the ‘Effects’ package, we isolated the factor of interest, ‘time of day’, while keeping the other elements (individual variance and day of experiment) constant (Fig. 2B). This procedure is akin to calculating an average day for an average individual, and allows calculating confidence intervals (CI). This plot shows that honeydew production was markedly rhythmic under LD conditions and peaked twice during day-time (ZT 0: mean 0.42, CI 0.34–0.51; ZT 9: mean 0.45, CI 0.37–0.54) and lowest activity at night time (ZT 18: mean 0.19, CI 0.13–0.27 drops). Activity appeared to be bimodal, though the confidence intervals overlap.

Under constant darkness the model with an interaction term (damping) was better suited (AIC = 3219) than the simpler model without damping (AIC = 3222). Honeydew excretion was rhythmic during the first days (day 1, peak at 20h after lights-off: mean 0.63, CI 0.51–0.75 drops; trough at 29h after lights-off: mean 0.24, CI 0.15–0.35 drops), but the only discernible peak damped quickly and disappeared after three days (Fig. 2C,D).

We decided to measure moulting mainly because it causes aphids to stop moving for longer time periods^33^, and thus disrupts the activity in the young aphids. By removing moulting individuals from the analysis of metabolism, we corrected for these gaps in activity. The disruption by moulting was more severe in our measures of locomotor activity. Therefore we considered metabolic activity of nymphs, but locomotor activity of adults for analysis of circadian rhythmicity.

### Locomotor activity: diurnal rhythms in LD and complex rhythms in DD

**Figure 3:**
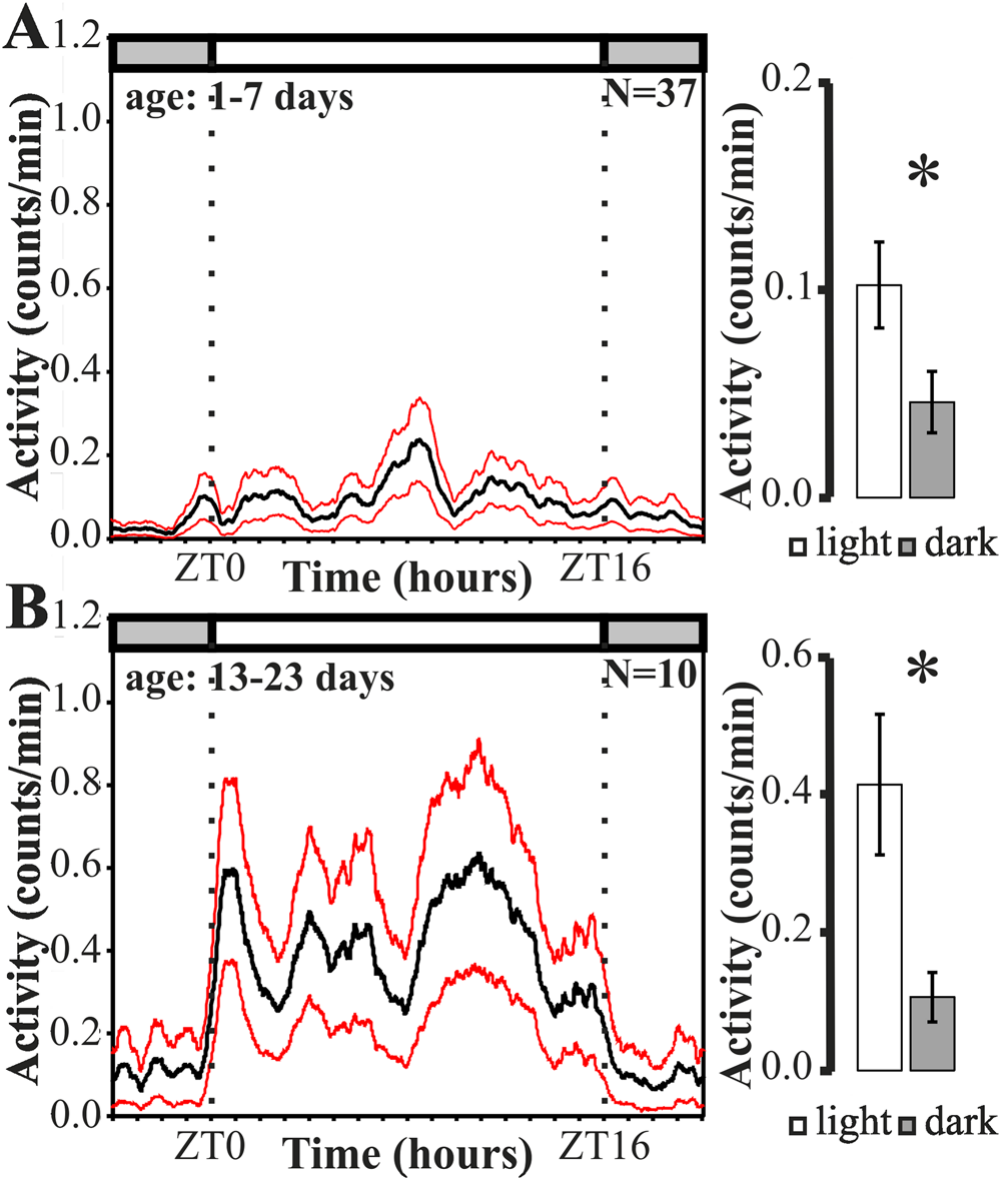
Diurnal activity profile in aphids. Average daily activity profile of nymphs (**A**) and adult (**B**) aphids measured by counting IR-beam passages (black line = mean activity, red line = standard error). Average day is calculated for day 1–7 for nymphs and day 13–23 for adult aphids. The light regime (LD 16:08) is indicated by the white (light phase) and grey (dark phase) bars above the graphs. Panels on the right: Mean activity of nymphs (1–7 days) (**A**) and adult (13–23 days) (**B**) aphids during light and dark phase.

**Figure 4:**
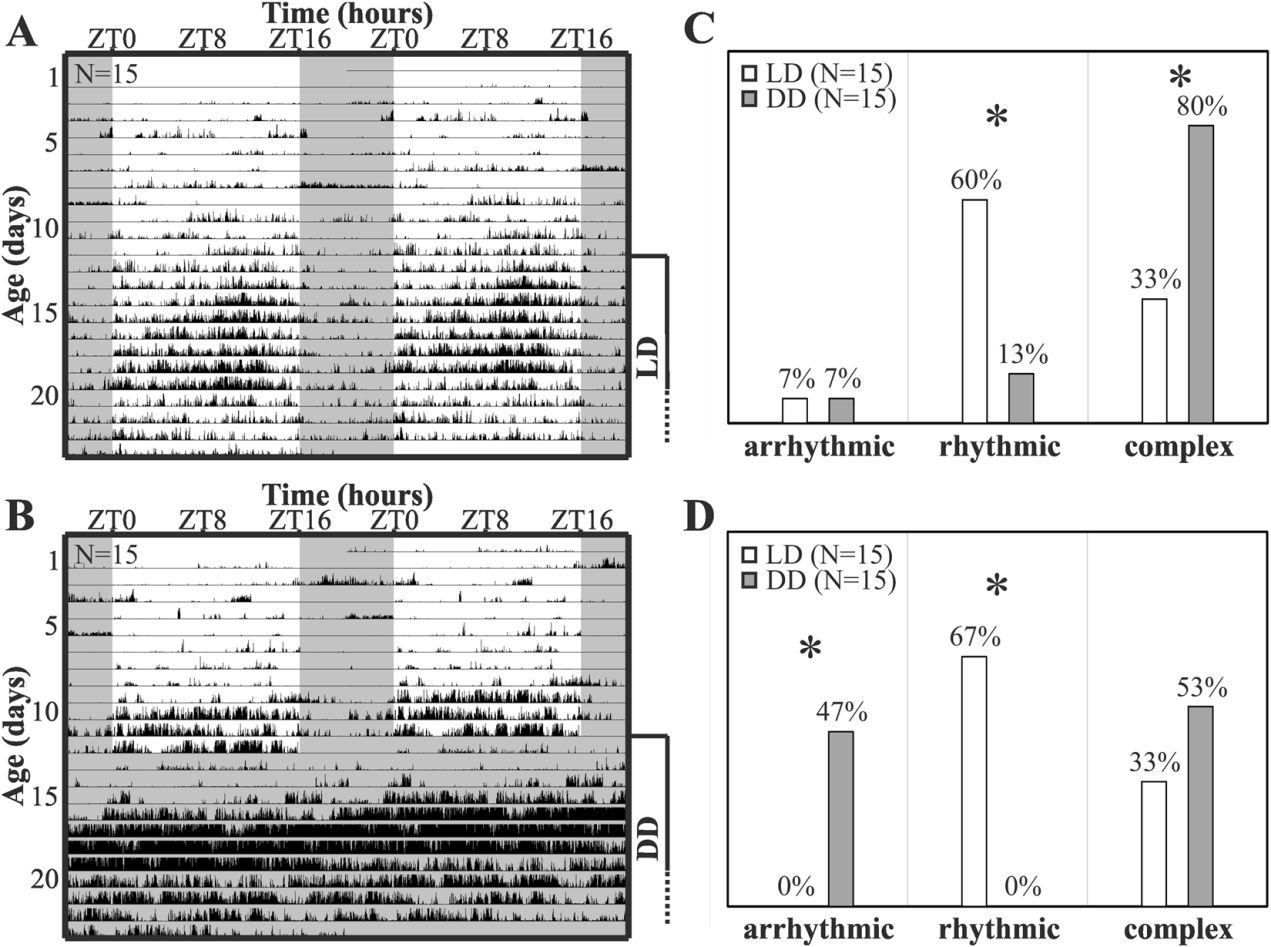
Locomotor activity rhythms of aphids under LD cycles and constant conditions. (**A**) Aphid activity plotted in average actograms (double plots of two consecutive days). Animals are monitored from nymphal stage L1 onwards for several days in LD 16:08 (light regime is indicated by the white (light phase) and grey (dark phase) background in the actograms). Activity levels day 20–23 are lower because a few aphids died in this period (in **A**: 4, in **B**: 1) (**B)** Like in **A** for the first 12 days in the setup, then aphids are released into constant darkness (DD). Percentage of rhythmic individuals analysed from day 13 onward for at least 5 consecutive days with activity (marked in A and B) in periodogram analysis with either Lomb-Scargle (**C**) or Chi-Square (**D**) method of testing rhythmicity.

We monitored locomotor activity rhythms of aphids during their whole live from nymphal stage L1 onward in a novel monitoring set up (Fig. 1). Both nymphs (age: 1–7 days; Fig. 3A) and adult aphids (age: 13–23 days; Fig. 3B) showed significantly higher average activity during the light phase than during the dark phase (p(nymphs) < 0.05, p(adult) < 0.05, paired Wilcoxon signed rank test). The activity patterns of the aphids were, however, disrupted by activity breaks lasting entire days during the first 9 days of their life (Fig. 4A/B), leading to lower activity levels in nymphs (0.08 ± 0.02 counts/min) than in adult aphids (0.31 ± 0.07 counts/min). The latter displayed two main activity bouts that were best visible in the average day, one after lights-on the other in the late light phase (Fig. 3B). In between the two activity bouts activity levels were lower.

While we detected diurnal rhythms in activity of adult aphids in LD, they appeared largely arrhythmic under constant conditions, at least in the average actogram (Fig. 4A/B). Nevertheless, averaging concealed the temporal and inter-individual variation, so we supplement our analysis with an individual-based approach. We found that many of the adult subjects (67%) showed no activity at all during the first three days after switching to constant conditions of DD at day 13 (Fig. 4B). We therefore selected periods of at least 5 continuous days with activity, and determined the percentage of rhythmic adults (from day 13 on) in LD and DD conditions via periodogram analysis (Lomb-Scargle (LS) method in Fig. 4C and chi-square (CS) method in Fig. 4D). In DD (N = 15) 0% (CS) to 13% (LS) of the aphids were rhythmic with a single peak in the periodogram and further 80% (CS: 53%) displayed complex rhythms with more than one significant predominant period. In LD (N = 15) 60% (67% with CS method) were rhythmic with one periodogram peak and 33% (both methods) showed complex rhythmicity. Although the percentage of arrhythmic individuals was significantly higher with the CS method in DD conditions (p < 0.01, Fisher’s exact test), the results of both analysis methods are similar, as the difference in rhythmic individuals between light treatments was significant in both tests (LS: p < 0.05; CS: p < 0.01, Fisher’s exact test).

## Discussion

By combining measurements of aphid honeydew production and locomotor activity, we find that aphid activity is bimodal under LD conditions. In constant darkness the rhythm persists initially, but then dampens quickly and individuals display complex rhythmicity.

We adapted a commercially available measurement system for locomotor activity of flies to monitor aphid rhythms with high data throughput. This novel monitoring method provides the first measurements of aphid locomotor activity rhythms independent of the host plant. It was sensitive enough to monitor adult aphids as well as nymphs, though the general activity level was lower than in most other insect species. For example *Drosophila melanogaster* ^34^, *Musca domestica* ^35^ and *Apis mellifera* ^36^ are approximately 10 times more active than the aphids in our locomotion setup. This finding can partly be explained by the unusual food source, which is known to compromise performance^17^. The aphids produced on average 2.5 honeydew drops per day, which is lower than the 4–8 drops/day reported in studies on plants^37^. On the other hand, different linden bug (*Pyrrhocoris apterous*) strains, which are of the same order (Hemiptera) and also feed by sucking, show similarly low activity levels^38^. Thus, the low locomotor activity levels might be related to the sap-sucking feeding behaviour.

Despite the low activity levels, aphids were clearly day active in both metabolism and locomotion. These results are in line with various studies that detected diurnal activity of aphids on plants^20,39,40^ as well as independently of the host plant^23^. Furthermore, aphids were shown to find their host plants more effectively in day light conditions^41,42^, indicating adaptation to a diurnal lifestyle.

The activity appeared bimodal in both behaviours with one peak around the time of lights-on and another one in the late light phase. Bimodal patterns of activity were described in a variety of different animals, for example fruit flies^43^, mosquitoes^44^, birds and rodents^45^, and have been linked to multi-oscillator systems^46^.

In the metabolic activity profile there is only one activity peak left in DD, which is probably due to a melting of the separate peaks like it has been observed in *Drosophila melanogaster* activity^43^. This peak damped quickly during the first 3–4 days in constant conditions. The observed damping of circadian rhythms could either be due to desynchronization of individual persisting rhythms or damping of individual rhythms, but the low number of honeydew drops and the resulting binomial data make it difficult to assess damping on the individual level. We thus incorporated inter-individual variation in a mixed-effects model to exclude population-level damping. The model is constrained in that the period length is fixed at 24 h, but we find it unlikely to affect our conclusions, given the 3-hour intervals of the data.

The results from locomotor activity are similar: As in the metabolic activity assay, the aphids showed clearly rhythmic behaviour in LD, and rhythmicity also persisted in DD, as the majority of individuals (LS: 93%, CS: 53%) remained rhythmic. This indicates that the behaviour is governed by the circadian clock. In contrast to the metabolic activity essay we found, however, no damping of rhythms, but instead the activity patterns transformed into complex rhythms in DD. Admittedly, most individuals in the locomotor assay were not active at all in the first few days in constant conditions, so we cannot exclude that the rhythmicity damped to some extent during the first days.

At the moment we can only speculate about the mechanisms that give rise to complex rhythms and individual level damping in DD. Complex rhythms are not unusual, and have been described before for example in mosquitoes^44^ and New Zealand Weta^47^. Lewis and co-authors proposed a two population model of circadian oscillators, driving different output rhythms. In this model different clock neuron populations in the oscillator are weakly coupled and oscillations driven by them become asynchronous in DD. Therefore different free running periods (FRPs) for the oscillator subgroups are detected, which creates complex rhythms as well as a damping in one circadian output rhythm, and eventually arrhythmicity on the individual and/or population level. We find this model appealing for aphid clocks, because it simultaneously explains the damping in one output and complex rhythms in the other output. Moreover, the model provides an explanation why some studies found clear circadian patterns^19^, whereas other studies with different outputs indicate hour-glass mechanisms^15^. Further studies on the molecular organization of the clock are needed to verify this model.

Moreover, since aphids produce honeydew during feeding and therefore metabolic and locomotor activity is directly connected, it is likely that similar mechanisms operate for these two outputs. Like the complex rhythms in locomotor activity might lead to dampened rhythmicity of the individual, damping of metabolic activity could occur at the individual level. Overall we found complex circadian rhythms in locomotion and quick damping in metabolism, and we argue that the aphid circadian clocks cannot drive strong activity rhythms.

Aphids played a central role in the development of the hourglass mechanism of the endogenous clock, which Lees postulated for the diapause induction of aphids^10,11^. Lees found no circadian involvement in diapause induction, causing him to question the coincidence models.

However, various aphid behaviours were found to be controlled by a circadian oscillator. For example clock gene homologues have recently been characterized, and their mRNA was shown to cycle^21,22^. On the behavioural level the circadian clock was shown to govern the release of aphid sex pheromone^19^, and fresh weight-gain and larviposition^20^. In the latter experiment, aphids were disrupted by shifting the light phase and the rhythms needed more than 3–4 days to re-entrain to the new conditions, which led the authors to the conclusion that this behaviour is governed by a circadian oscillator.

As an alternative model to the hour-glass model, the circadian oscillator model with damping rhythms^48^ can simulate the “hour-glass reaction” observed in diapause induction of aphids. However, although this model fits to the data of Lees, only recently actual damping of circadian rhythms in aphids has been demonstrated^21^. In this study the cycling of mRNA levels extracted from whole mount aphid brains appeared to rapidly dampen in constant conditions. Although whole mount extracts provide only a population perspective and the animals were not kept independently of the host plant, these results point to a damped circadian clock in aphids. Our study adds the analysis of output rhythms independently of the host plant and supports the model of an oscillator driving damping circadian rhythms in two points.

Firstly we observe circadian oscillations in behaviour and metabolic activity in DD, which would not be the case if the aphid endogenous clock would have an hourglass mechanism. Secondly, we see a clear damping of activity rhythms in the first few days in constant conditions, at least in metabolic activity. Hence, our study provides a first empirical evidence for a damping oscillation in aphid activity.

Though anticipating daily reoccurring events is an advantage, an oscillator that drives strong circadian rhythms over a long time is potentially less flexible in resetting to seasonal changes. There are environmental situations on earth that require organisms to adapt to extreme deviations from daily rhythms. For example reindeer living in polar regions have been found to be arrhythmic during extreme illumination conditions of the arctic summer and winter^49–51^. This adaptation enables those animals to optimally use the short season for gaining resources and hence show a strong seasonal rhythm in locomotor and metabolic activity. A similar seasonal adaptation of the circadian system has been found in *Drosophila* species, *D*. *montana*, *D*. *ezoana and D*. *littoralis*, living in Northern Europe^52–54^. Unlike *D*. *melanogaster*, which has shallow photoperiodic diapause induction^55^, these flies are exposed to extreme photoperiods and evolved a very robust diapause induction, while they have poor circadian activity rhythms in constant conditions. We can certainly draw here parallels to our results on aphid rhythmicity. Like the polar reindeer or the Northern *Drosophila* species, aphids exhibit very weak circadian rhythms, while the seasonal response to shortening photoperiod is highly robust.

The weak circadian response in aphids might be the reason why most interest so far was put on the investigation of photoperiodism while the circadian clock remained widely unattended. But this way a complete picture of clock output characteristics was missing. Our study demonstrates that the investigation of different outputs, by combining expert knowledge of different disciplines is important to gain a better understanding about properties of the aphid circadian clock. There is an ongoing discussion whether the circadian clock and the annual photoperiodic clock are two independent timing systems or if they are at least partially the same^5,6^. While our study offers no direct causal link between damping activity rhythms and photoperiodism, it provides the tools of measuring two different clock outputs that could be used in future experiments with varying photoperiods. Further investigation of the circadian clock in aphids, also on the molecular level, might provide us with a deeper understanding of the potential involvement of the circadian system in seasonal timing.

## Electronic supplementary material

Dataset1A

Dataset1B

Dataset2A

Dataset2B

Dataset2C
