# Supplementary material for: A damping circadian clock drives weak oscillations in metabolism and locomotor activity of aphids (Acyrthosiphon pisum)
Source: Sci Rep. 2017 Nov 2;7:14906. doi: 10.1038/s41598-017-15014-3 (PMC5668311; doi:10.1038/s41598-017-15014-3)
Supplement: Supplementary file 4 — Dataset2B [file 41598_2017_15014_MOESM4_ESM.doc]

The dataset metabolic.txt belongs to the publication "A damping circadian clock drives weak oscillations in metabolism and locomotor activity of aphids (*Acyrthosiphon pisum*)" by Katharina Beer, Jens Joschinski, Alazne Arrazola Sastre, Jochen Krauss and Charlotte Helfrich-Förster.

We measured rhythms in honeydew excretion on an artificial diet by inspecting individually reared aphids every three hours, and noting whether drops were produced.

Measurement: consecutive numbering of measurement round (each three hours apart)

Person: 4 people were involved in the measuring procedure. These are the first names of the 4 persons.

Day: Consecutive numbering of day of experiment. Day 2 starts with measurement 7.

Time: Time of day in 24 h- format (NOT zeitgeber time). 8 = ZT 0 (lights-on).

Treatment: Light-dark conditions (LD) with 16 hour photophase, or constant darkness (DD).

ID: consecutive numbering of individual petri dishes (each with one aphid)

drops: Occurence of honeydew drops (1) or no drops (0).In 96.6% of all cases, (1) equals a single honeydew drop

Exuviae: Total number of exuviae counted.All surviving aphids were in last larval stage (L4) at the end of the experiment, so 3 exuviae were expected in the petri dishes.

Morph: After the experiment the aphids were either identified as winged (F) or wingless (U). Some aphids died before reaching the fourth larval stage, and could thus not be identified

new_ex: This column denotes whether a new exuvia has been produced. In contrast to the column "Exuviae", this column is logical (0 or 1) and only counts new exuviae (to identify aphids that currently moult).
